# Molecular interactions between the olive and the fruit fly *Bactrocera oleae*

**DOI:** 10.1186/1471-2229-12-86

**Published:** 2012-06-13

**Authors:** Giandomenico Corrado, Fiammetta Alagna, Mariapina Rocco, Giovanni Renzone, Paola Varricchio, Valentina Coppola, Mariangela Coppola, Antonio Garonna, Luciana Baldoni, Andrea Scaloni, Rosa Rao

**Affiliations:** 1 Dipartimento di Scienze del Suolo, Pianta, Ambiente e Produzioni Animali, Universita’ degli Studi di Napoli Federico II, Via Università 100, Portici, Napoli, 80055, Italy; 2 Istituto di Genetica Vegetale, Consiglio Nazionale delle Ricerche, Via della Madonna Alta 130, Perugia, 06128, Italy; 3 Dipartimento di Scienze per la Biologia, la Geologia e l’Ambiente, Universita’ del Sannio, Via dei Mulini 59/A, Benevento, 82100, Italy; 4 Istituto per il Sistema Produzione Animale in Ambiente Mediterraneo, Consiglio Nazionale delle Ricerche, Via Argine 1085, Napoli, 80147, Italy; 5 Dipartimento di Entomologia e Zoologia Agraria “F. Silvestri”, Universita’ degli Studi di Napoli Federico II, Via Università 100, Portici, 80055, Italy

**Keywords:** *Olea europea*, Pest, SSH, Proteomics, Defence, Fruit fly

## Abstract

**Background:**

The fruit fly *Bactrocera oleae* is the primary biotic stressor of cultivated olives, causing direct and indirect damages that significantly reduce both the yield and the quality of olive oil. To study the olive-*B. oleae* interaction, we conducted transcriptomic and proteomic investigations of the molecular response of the drupe. The identifications of genes and proteins involved in the fruit response were performed using a Suppression Subtractive Hybridisation technique and a combined bi-dimensional electrophoresis/nanoLC-ESI-LIT-MS/MS approach, respectively.

**Results:**

We identified 196 ESTs and 26 protein spots as differentially expressed in olives with larval feeding tunnels. A bioinformatic analysis of the identified non-redundant EST and protein collection indicated that different molecular processes were affected, such as stress response, phytohormone signalling, transcriptional control and primary metabolism, and that a considerable proportion of the ESTs could not be classified. The altered expression of 20 transcripts was also analysed by real-time PCR, and the most striking differences were further confirmed in the fruit of a different olive variety. We also cloned the full-length coding sequences of two genes, Oe-chitinase I and Oe-PR27, and showed that these are wound-inducible genes and activated by *B. oleae* punctures.

**Conclusions:**

This study represents the first report that reveals the molecular players and signalling pathways involved in the interaction between the olive fruit and its most damaging biotic stressor. Drupe response is complex, involving genes and proteins involved in photosynthesis as well as in the production of ROS, the activation of different stress response pathways and the production of compounds involved in direct defence against phytophagous larvae. Among the latter, trypsin inhibitors should play a major role in drupe resistance reaction.

## Background

The olive fruit fly *Bactrocera oleae* (Rossi) (Diptera: Tephritidae) is the most harmful pest of olives worldwide [1]. Primarily known as a cause of significant yield loss in almost all of the countries of the Mediterranean Basin (where the major olive and oil producing countries are located), this monophagous pest is currently also present in new areas of cultivation, such as South Africa and North and Central America [2,3]. The olive fruit fly is able to reduce crop yield in several ways [1]. Adult females injure drupes through their oviposition on the ripening fruits. The newly hatched larva will grow as a fruit borer, excavating a tunnel in the mesocarp until pupation. Larval feeding causes yield loss primarily by pulp consumption and inducing premature fruit dropping. Additionally, infested fruits present an alteration of their organoleptic features that makes them unsuitable for direct consumption, transformation or pressing [4]. Although the availability and quality of host fruits, along with climate, represent important triggers of *B. oleae* outbreaks, it has been estimated that the average crop loss is in the range of 5–30% of the total olive production, even with intense chemical control measures [3,5]. Conventional management methods rely on insecticide applications to control the fly after monitoring the adult population [1]. Unfortunately, similarly to many other pests, populations of *B. oleae* have acquired insensitivity to insecticides [6,7]. Moreover, classical biological control programs have not been successful, particularly in that they fail to consistently provide adequate levels of control across the range of climates and of cultivated olive varieties [1].

Despite the severe impact on yield, comprehensive studies on the olive response and on resistance mechanisms to the fruit fly are still lacking. Olive cultivars differ in the degree of susceptibility to fruit fly infestation [1], but the factors underlying this trait are still controversial [8,9]. A strong tolerance, defined mainly by assessing the severity of the infestation, has been reported in some cultivated varieties [1]. However, even the so–called “resistant” cultivars may suffer considerable attacks under intense infestation pressure [10]. It is likely that the differential susceptibility to the fruit fly may involve a number of morphological, physiological and phenological parameters, which include mechanical obstruction, fruit composition and the amount of chemicals involved in plant direct and indirect defence [8,11,12]. Unfortunately, studies aimed at the description of the molecular response of the olive to *B. oleae* are also much needed to understand the mechanisms and the players of olive defence, eventually improving stress resistance, increasing yield and facilitating the molecular selection of olive varieties more suitable for Integrated Pest Management.

To gain a more thorough understanding of the consequences of the olive–fruit fly interaction, we studied the molecular response of the fruits at the transcriptional and proteomic levels. Due to the limited information on the olive genome, a PCR approach on subtracted cDNA libraries was used. The PCR–based Suppression Subtractive Hybridisation (SSH) technique was developed for a sensitive comparison of mRNA expression patterns between two cDNA populations [13]. This methodology has been successfully exploited to analyse plant responses to biotic or abiotic stress and changes between different developmental stages or tissues [14-18]. Although the SSH method has been widely used in the animal, prokaryotic and human fields, it is particularly useful for species that lack genomic data [19]. In parallel, a bi-dimensional electrophoresis analysis of protein extracts was used to identify specific proteomic changes in drupes with larval feeding tunnels. Gel-based proteomic studies have been extensively used to investigate protein expression changes in plant tissues during responses to biotic or abiotic stress and to highlight molecular signatures in genotypes with higher levels of resistance to insects or fungi [20-24]. Our transcriptomic and proteomic analyses allowed us to reveal the molecular bases and related signalling pathways induced in the interaction between olive and its most damaging biotic pest.

## Results

### Construction of the subtracted library and sequence analysis

A subtracted library was constructed to identify olive genes whose expression is affected by *B. oleae* infestation. The cDNA library was obtained using RNA from fruits with larval feeding tunnels as *tester* and from undamaged fruits as *driver.* Blue/white selection and restriction digestion identified 590 recombinant colonies out of 1,180. After filtering for size by restriction analysis (>200 bp), the recombinant plasmids were sequenced and the clones with low information content were removed. The average length of the 196 cloned olive sequences was 303 bp, from a minimum of 69 bp to a maximum of 766 bp. To obtain unique sequences (unigenes), we performed an assembly using the CAP3 program, which identified 87 singletons and clustered the remaining 111 sequences in 33 contigs, made up of 2 to 22 overlapping ESTs. The resulting non-redundant unigene dataset, following an *in silico* automated translation, was compared against available databases to find similarities with known sequences. Only three clones matched already available olive sequences. Matches with e-values lower than 10e-03 were used to assign a putative function to the transcripts. Overall, 39.2% of the unigenes putatively code for proteins with significant similarity to annotated proteins in other organisms; these unigenes were named based on the homology (Additional file 1). The remaining 73 unigenes (60.8%) were considered to be functionally unidentified. Unigenes with a blastX e-value higher than 10e-3 were then compared with nucleotide databases. Fifty-two sequences revealed a significant similarity (e-value lower than 10e-3). Specifically, 27 (respectively 2) ESTs were annotated, choosing as the search set the non-redundant nucleotide collection (respectively the Expressed Sequence Tags collection) at NCBI. A significant similarity for another 23 clones was found by analysing the olive transcribed sequences at the OLEA EST db. All of these sequences are listed in Additional file 2. The proportion of sequences that were not annotated may be explained by the relatively small average length of the SSH fragments, the presence of fragments including UTR regions, which typically correspond to less-conserved regions of genes, or both. These two features are likely to be introduced by the subtraction technique [25,26], which favours the cloning of relatively short fragments with lower degrees of conservation. The average length of the non-described sequences significantly differed from that of the annotated entries (*t*-test; *p* < 0.01). However, over 71% of the SSH clones whose putative translation product could not be annotated nevertheless showed similarity with other plant transcripts. Approximately half of these clones found a match exclusively in the Olea EST database, suggesting that the lack of a functional annotation relative to a set of inducible genes, is also due to the existence of sequences that are specific to the olive tree.

**Figure 1:**
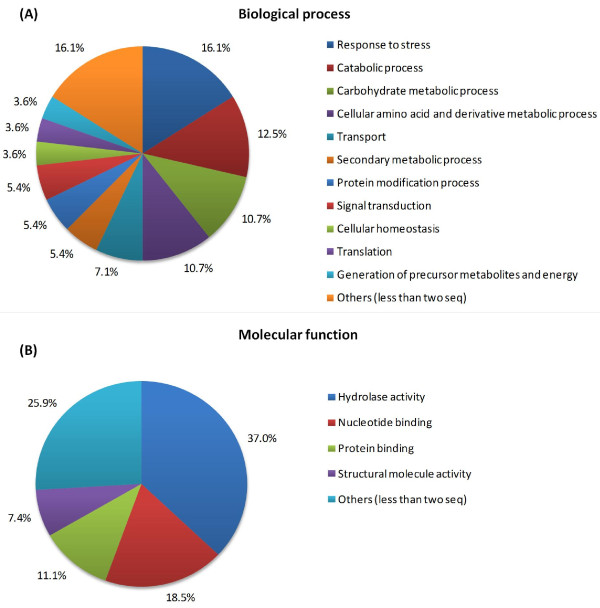
**Distribution of biological process (a) and molecular function (b) terms of the 47 annotated unigenes following GO classification (73 sequences were not annotated).** To provide a multi-level summary of the GO functions using a prepared plant GO set, we performed a Plant GoSlim annotation with a cut-off of two sequences. The category “others” includes DNA metabolic process, cell differentiation, response to biotic stimulus, response to abiotic stimulus, anatomical structure morphogenesis, cell death, flower development, growth, and response to endogenous stimulus for “biological process”. For “molecular function” the category “others” includes kinase activity, receptor activity, carbohydrate binding, DNA binding, enzyme regulator activity, transporter activity, and transferase activity

Gene Ontology analysis of the non-redundant unigene collection was performed using the Blast2GO software, considering the limited information available regarding the olive genome. The sequences were classified into two ontology categories, namely, “Biological process” and “Molecular function” (Figure 1). Interestingly, in the category “Biological process”, the most frequent entry was “Response to stress”, followed by “Catabolic process”. The most frequent “Molecular function” was hydrolase activity [27]. Among the annotated sequences in the SSH library, we identified several ESTs whose homologues in other species are associated with plant responses to biotic stress, such as proteins of the hydrolase family and proteinase inhibitors.

### Expression analysis of ESTs involved in plant defence

**Figure 2:**
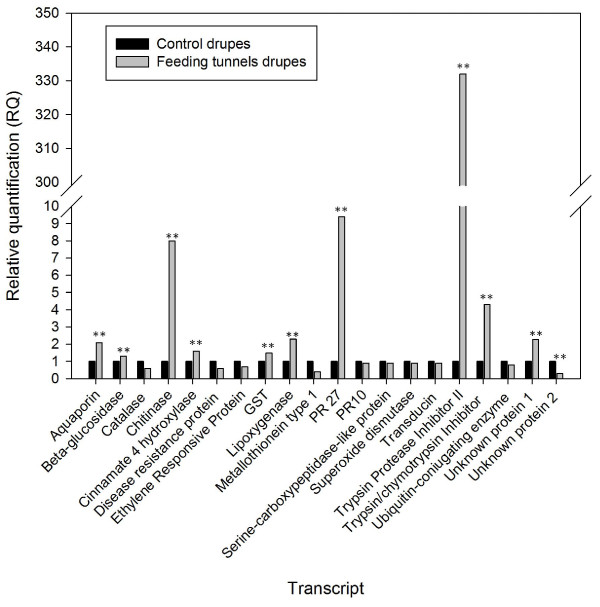
**Real-time PCR analysis of the expression levels of transcripts putatively involved in defence against larval feeding.** The genes are listed in Table 1**.** RNA was isolated from drupes of the cultivar ‘Moraiolo’. The graph displays the relative quantity (RQ) for each target gene in the control (black bars) and infested drupes (grey bars), shown on a linear scale relative to the calibrator (control drupes). Asterisks indicate significant difference compared to control (*p* < 0.01)

The *B. oleae*-inducible expression of a selection of unigenes was also investigated by quantitative real-time PCR (qRT-PCR), as a verification based on an independent experimental method and material [28]. To this end, we selected from the library 18 unigenes representative of different biological processes, such as abiotic and biotic defence response, signal transduction, phytohormone signalling and transcriptional regulation. Furthermore, we included in the analysis two transcripts putatively coding for unclassified proteins (Table 1). The qRT-PCR experiments were performed using RNAs isolated from infested and control drupes of the cultivar ‘Moraiolo’ harvested in a different year. The real-time RT-PCR data indicated that ESTs exhibiting significant similarities to Trypsin Protease Inhibitor II, Trypsin/chymotrypsin Inhibitor, Pathogenesis Related protein 27 (PRp27) and chitinase I were the most highly inducible sequences by larval feeding (Figure 2). The high inducibility of TPI II was also confirmed in drupes of a different cultivar, ‘Leccino’ ( 3).

**Table 1:** Genes selected for validation of expression by quantitative RT-PCR and their putative functions

**Gene name**	**ID**	**Acc. Number**	**Best similarity [Species]; acc. number**	**e-value**	**Gene Ontology terms**
Aquaporin	148	JQ711526	Aquaporin PIP2 [Vitis vinifera]; ABN14353.1	3,60E-19	F: water channel activity
					P: response to abscissic acid stimulus
Beta-glucosidase	C17	AAL93619.1	Beta-glucosidase [Olea europaea]; AAL93619.1	1,08E-56	F:hydrolase activity
					P:carbohydrate metabolic process
Catalase	119	ABS72010.1	Catalase [Olea europaea]; ABS72010.1	5.60E-23	F:catalase activity
					P:response to oxidative stress
Chitinase	2	JN696113	Extracellular chitinase, class I [Vitis vinifera]; XP_002269972.1	7,55E-25	F: chitinase activity
					P:defense response to fungus
Cinnamate 4 hydroxylase	C32	JQ711532	Trans-cinnamate 4-hydroxylase [Populus tremuloides]; ABF69101.1	5,56E-39	F: trans-cinnamate 4-monooxygenase activity
					P:response to wounding
Disease resistance protein	C4	JQ711509	Disease resistance response protein 206 [Zea mays]; NP_001149569.1	9,94E-09	P:defense response
Ethylene Responsive Trascription Factor	53 F	JK784503	Ethylene-responsive transcription factor (ERF) [Olea europaea]; OLEEUCl001226:Contig3	1,00E-100	F: DNA binding
					P: ethylene mediated signaling pathway
GST	59-P	JQ711516	Glutathione S-transferase [Hyoscyamus muticus]; P46423.1	2.87E-21	F: glutathione transferase activity
					P: defense reponse
Lipoxygenase	301-P	EU513351	Lipoxygenase [Arabidopsis thaliana]; CAC19365.1	7.00E-13	F: lipoxygenase activity
					P: response to wounding
Metallothionein type 1	52-L	JQ711520	Metallothionein-like protein [Pimpinella brachycarpa]; AAC62510.1	2,35E-10	F: copper ion binding
					P: cellular copper ion homeostasis
PRp27	C2	JN696114	NtPRp27 [Nicotiana tabacum]; BAA81904.1	1,07E-78	P:defense response
PR 10	C6	JQ711524	Trypsin chymotrypsin inhibitor [Lens culinaris]; CAR47883.1	1,10E-36	F: serine-type endopeptidase inhibitor
					P: defense response
Serine-carboxypeptidase-like protein	C29	JQ711528	Serine carboxypeptidase [Ricinus communis]; EEF40335.1	1,22E-14	F: serine-type carboxypeptidase activity
					P: proteolysis
Superoxide dismutase	3-F	JK784468	Cu/Zn super-oxide dismutase (Ole e 5 allergen) [Olea europaea]; AJ428575.2	2,09E-111	F: superoxide dismutase activity
					P: response to oxidative stress
Transducin	109-N	JQ711533	Transducin family protein [Arabidopsis lyrata]; EFH62484.1	4,52E-22	F:nucleotide binding
Trypsin Protease Inhibitor II	99-N	JQ429796	Proteinase inhibitor type-2 [Solanum lycopersicum]; CAA64416.1	1.14E-12	F: serine-type endopeptidase inhibitor
					P: defense response
Trypsin/chymotrypsin Inhibitor	93	JQ429797	Trypsin chymotrypsin inhibitor [Lens culinaris]; CAR47883.1	1,10E-36	F: serine-type endopeptidase inhibitor
					P: defense response
Ubiquitin-coniugating enzyme	C13	JQ429798	Ubiquitin-conjugating enzyme E2 [Ricinus communis]; XP_002523377.1	7,64E-46	F: ubiquitin-protein ligase activity
					P:response to iron ion
Unknown protein 1	51-F	JQ711535			
Unknown protein 2	C12	JQ711536			

The GO term associations for the gene product were retrieved and selected from the best blast matches at the AmiGO website (http://amigo.geneontology.org). F: molecular function. P: biological process.

### Identification of two full-length coding sequences and stress-response transcriptional analysis

As part of this study, we identified the full-length coding sequences of two genes highly inducible by larval feeding and further characterised their involvement in plant-insect interactions. The cDNA fragments of PRp27 and chitinase were used as starting point for 5′ and 3′ RACE-PCRs. The assembly of the rescued cDNA fragments allowed us to identify the full-length coding sequences of 792 and 687 bp for the olive chitinase and PR27 genes, respectively. Moreover, the 3′ UTR and part of the 5′ UTR were also cloned. These genes were named Oe-Chitinase I (Acc. Num. JN696113) and Oe-PRp27 (Acc. Num. JN696114).

**Figure 3:**
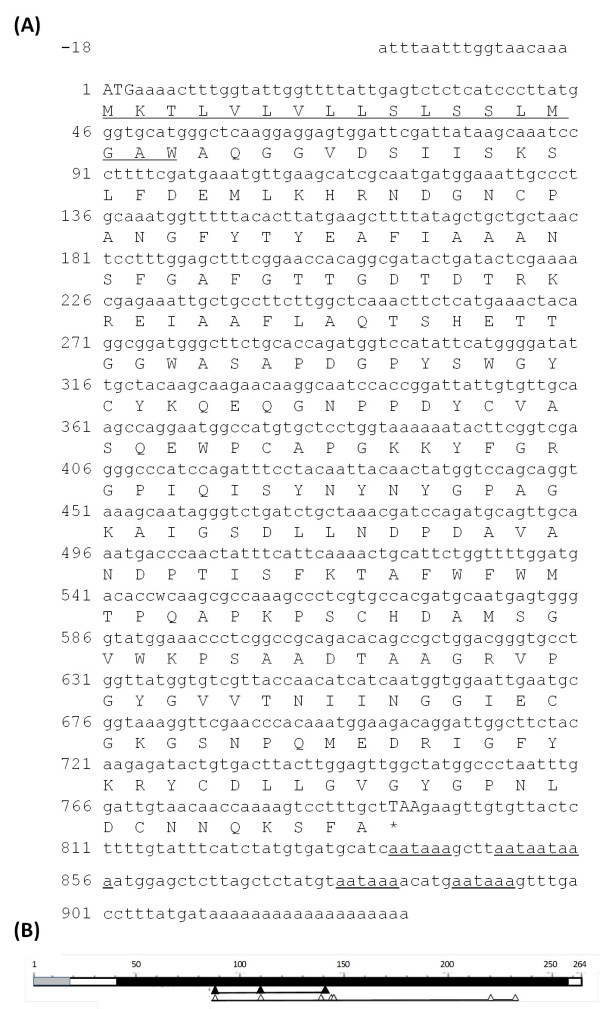
**Primary structure of Oe-Chitinase I. (a)** The nucleotide sequence of Oe-Chitinase I with its deduced amino acid sequence. The start and stop codons are in capital letters. The putative signal peptide at the N-terminus is doubly underlined. The five putative poly(A) additional signals are underlined. **(b)** A schematic representation of the Oe-Chitinase I protein showing the signal peptide (grey area), the catalytic domain of the chitinase-glycohydrolase 19 family (black area) and the positions of catalytic residues (black triangles) and putative sugar binding sites (white triangles)

**Figure 4:**
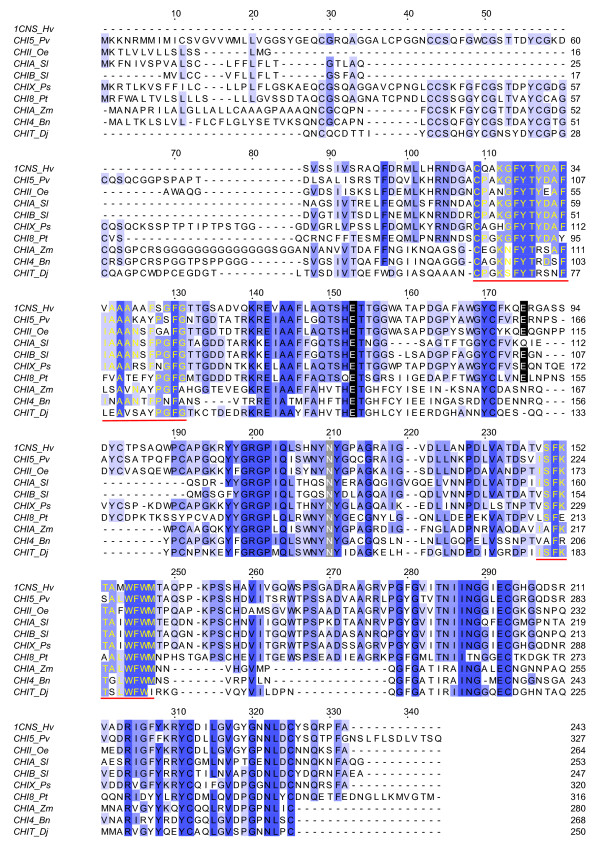
**Multiple alignment of the amino acid sequences of representative plant chitinases chosen among those with a structure-link.** The residues conserved in more than 80%, 60% and 40% of the proteins examined are indicated by dark blue, violet and light blue backgrounds, respectively. The two catalytic amino acid residues of family 19 chitinases are indicated by black boxes. The conserved motifs of the chitinase 19 family are underlined in red. The residues in grey boxes are related to the enzymatic activity. The chitinases used are CHI_I_Oe from olive (JN696113, *Olea europea*), 1CNS_Hv from barley (1CNS_A, *Hordeum vulgare*), CHI5_Pv from bean (P36361, *Phaseolus vulgaris*), AGI_Ud from stinging nettle (P11218, *Urtica dioica*), CHIT_DJ from Japanese yam (P80052, *Dioscorea japonica*), CHIA_Zm from maize (P29022, *Zea mays*), CHI4_Bn from rape (Q06209, *Brassica napus*), CHIX_Ps from pea (P36907, *Pisum sativum*), CHIA_Sl from tomato (Q05539, *Solanum lycopersicum*), CHIB_Sl from tomato (Q05540, *Solanum lycopersicum*), and CHI8_Pt from black cottonwood (P16061, *Populus balsamifera* subsp. *trichocarpa*)

The assembled cDNA sequence of Oe-Chitinase I was 947 bp with an ORF coding for a polypeptide containing 264 amino acids with a theoretical pI of 5.61 (Figure 3). This protein product has the highest sequence identity (81%) with chitinase 1b of *Vitis vinifera* (Acc. Num. CAC14015) and contains a conserved domain typical of the Chitinase 19 superfamily. The lack of an N-terminal chitin-binding domain (a 30- to 43-residue motif organised around a conserved four-disulphide core) implies that Oe-Chi I belongs to the class IB/II. Proteins belonging to the Chitinase 19 family are enzymes whose function in plants is primarily associated with the defence response to fungal and insect pathogens. Bioinformatics revealed the presence of a 19-amino acid N-terminal Signal Peptide and the lack of a vacuole targeting signal, suggesting that Oe-Chi I is secreted into the extracellular space. Multiple alignment of the amino acid sequence to other plant chitinases revealed the presence of several conserved residues, most notably three catalytic residues, four sugar-binding sites and two signature patterns of the Chitinase 19 family (Figure 4).

**Figure 5:**
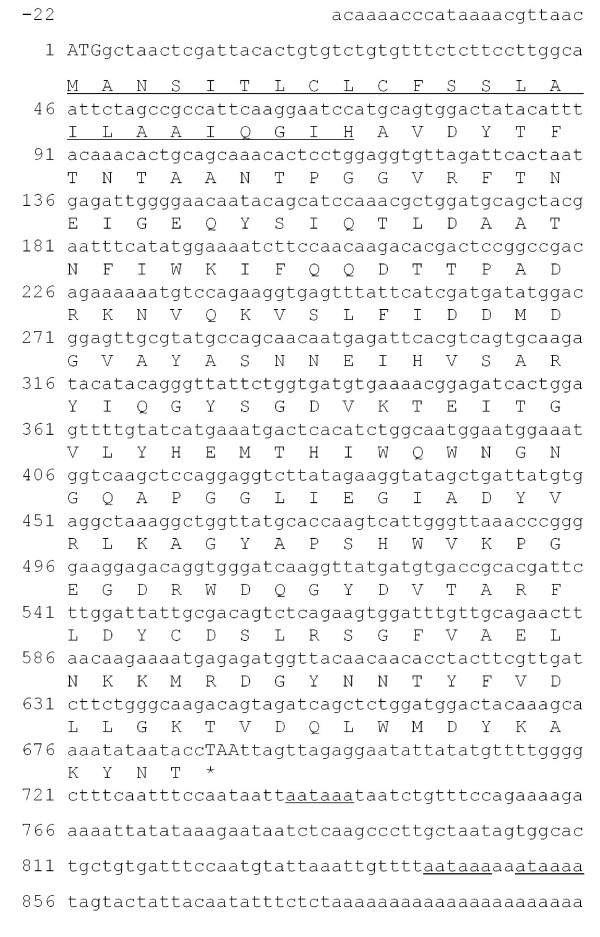
**Primary structure of Oe-PRp27.** The nucleotide sequence of Oe-PRp27 with its deduced amino acid sequence. The start and stop codons are in capital letters. The putative signal peptide at the N-terminus is doubly underlined. The putative poly(A) additional signals are underlined

**Figure 6:**
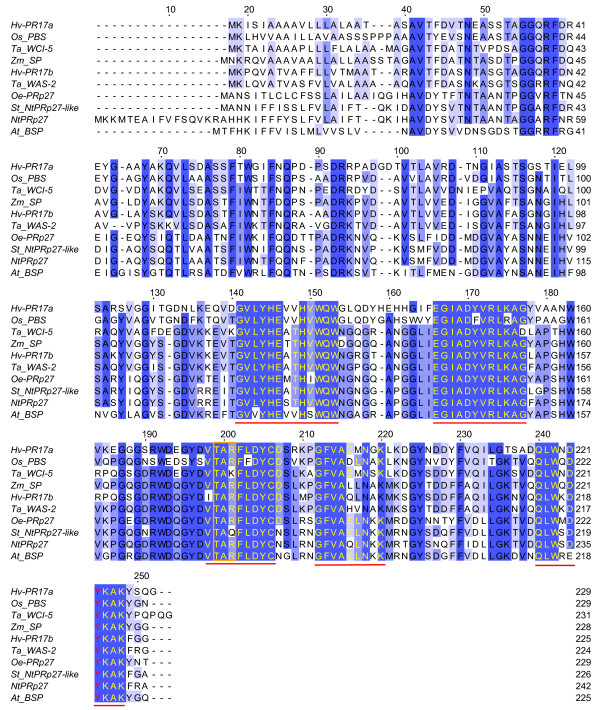
**Multiple alignment of the amino acid sequences of members of the PRp17 family.** The residues conserved in more than 80%, 60% and 40% of the proteins examined are indicated by dark blue, violet and light blue backgrounds, respectively. The five conserved motifs of the PR17 family are underlined in red. The conserved protein kinase C phosphorylation site (TXR/K/Q) is in an orange rectangle and the Tyr residue with the potential to act as a donor is shown in red. The proteins used are Hv-PR17a from barley (Y14201, *Hordeum vulgare*), Os_PBS from rice (NP_001064926, *Oryza sativa Japonica*), Ta_WCI-5 from wheat (AAC49288, *Triticum aestivum*), Zm_SP from maize (B6TDW7, *Zea mays*), Hv-PR17b from barley (Y14202, *Hordeum vulgare*), Ta_WAS-2 from wheat (AF079526_1, *Triticum aestivum*), St_NtPRp27-like from potato (Q84XQ4, *Solanum tuberosum*), Oe-PRp27 from olive (JN696114, *Olea europea*), NtPRp27 from tobacco (Q9XIY9, *Nicotiana tabacum*), and At_BSP from thale cress (AF345341, *Arabidopsis thaliana*)

The assembled cDNA sequence of Oe-PRp27 was 926 bp with an ORF coding for a polypeptide containing 229 amino acids (Figure 5). This protein product has the highest sequence identity (77%) with NtPRp27 from *Nicotiana tabacum* (Acc. Num. BAA81904). The predicted Oe-PRp27 protein contains a basic secretory protein (BSP) domain, which is believed to be typical of proteins involved in plant defence mechanisms against pathogens. Bioinformatic analysis indicated the presence of a 24-amino acid N-terminal Signal Peptide. Multiple sequence alignment revealed the presence of five regions that are highly conserved among members of the PR17 family of pathogenesis-related plant proteins [29] (Figure 6).

**Figure 7:**
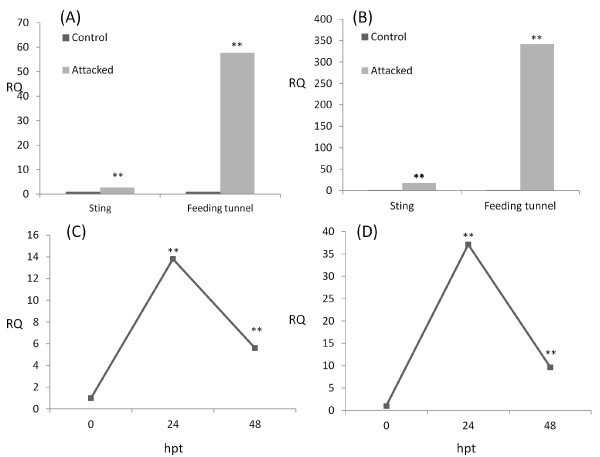
**Real-time PCR analysis of the relative expression levels of the Oe-Chi I and Oe-PRp27 genes in relation to biotic and mechanical stress.** RNA was isolated from drupes of the ‘Leccino’ cultivar. The graph displays the relative quantity (RQ) for each target gene (grey bars), shown on a linear scale relative to the calibrator (control drupes; white bars). Asterisks indicate significant difference compared to control (*p* < 0.01). **a)** The expression level of Oe-Chi I in drupes with oviposition punctures or larval feeding tunnels. **b)** The expression level of Oe-PRp27 I in drupes with oviposition punctures or larval feeding tunnels. **c)** A time-course of the expression level of Oe-Chi I in drupes following mechanical damage, at 0 (control), 24 and 48 h following treatment (hpt). **d)** A time-course of the expression level of Oe-PRp27 I in drupes at 0 (control), 24 and 48 h following treatment (hpt)

The transcriptional profile of these two genes was further investigated. We analysed the relative gene expression in fruits with *B. oleae* oviposition punctures or with feeding tunnels. Furthermore, we determined their response to wounding, one and two days after treatment (Figure 7). Considering that intraspecific variations in herbivory-induced signalling events and secondary metabolites exist among different plant populations [30], this work was performed in a different cultivated variety, ‘Leccino’. Our results indicated that both Oe-Chitinase I and Oe-PR27 transcripts accumulate in response to fruit fly punctures and confirmed their strong activation in drupes with larval feeding tunnels. Furthermore, the wounding experiments showed that these genes are also inducible by mechanical damage. Both genes had their higher transcriptional level at 24 h after treatment and interestingly, at this time-point, their level of expression of was similar to that recorded in olives punctured by the fruit fly. These data also suggest that other factors, most likely those related to the feeding habit of the larva, should be present to achieve the full induction of both genes in drupes with feeding tunnels.

### Identification of differentially expressed proteins

**Figure 8:**
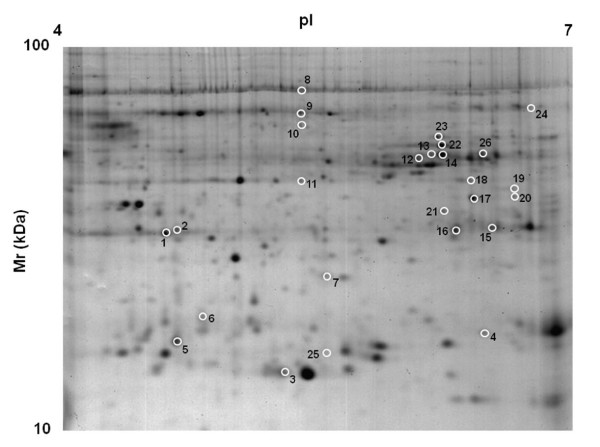
**2D proteomic map of drupes from control olives after staining with colloidal Coomassie G250.** The numbers indicate protein spots showing statistically significant differences with *Bactrocera oleae*-infested fruits. These spots are described in the additional file 4

A proteomic approach was used to ascertain the qualitative and quantitative modifications in the protein expression profile of olive fruits due to larval feeding. Protein extracts were prepared from infested and control fruits and subjected to 2-DE analysis. Software-assisted densitometric analysis of the resolved gels allowed a comparison of the respective proteomic repertoires. A representative Coomassie-stained gel from the control olives is shown in Figure 8. The average proteomic maps showed 578 (control fruits) and 498 (insect-infested fruits) spots, respectively, with a 77% similarity. The statistical evaluation (*p* < 0.05) of the relative spot densities detected 26 spots as differentially present in fruits subjected to larval attack, with at least a twofold difference with respect to the control. Eight spots showed increased abundance levels in insect-infested fruits, whereas the remainder exhibited the opposite trend. These spots were excised from the gels, digested with trypsin and subjected to nanoLC-ESI-LIT-MS/MS analysis. A database search with data deriving from the MS experiments allowed a positive identification of 23 spots. The list of the identified proteins is reported in the additional file 4, together with their quantitative variations. For 19 spots, MS analysis demonstrated the occurrence of a single protein component within the analysed sample. Conversely, multiple polypeptide species (2–3 in number) were detected in each of the remaining 4 spots, as result of their concomitant electrophoretic migration. The identified sequences were compared against the available sequence databases to find similarities with known plant proteins; the entries with the highest scores are reported in the additional file 4. According to GO classification, the differentially expressed proteins are primarily involved in carbohydrate metabolism, redox processes and defence responses.

## Discussion

In the SSH library approximately 40% of the unigenes identified in the SSH library could be functionally annotated. Although a comparison between different works is difficult because of the increasing number of sequences in the databases, this proportion is lower than those reported in similar works about model plant species [16,18], yet it is similar to other reports on species whose genomes have not yet been sequenced [25,31]. Interestingly, the functional characterisation of the library indicated a higher representation of ESTs involved in the plant response to stress, including those related to biotic stress, such as wounding and pathogen attack, or abiotic stress, such as high or low temperature, drought and NaCl. Moreover, we identified transcripts involved in the production, signal transduction or response to hormones and molecules (e.g., jasmonic acid and ROS) that are related to plant resistance to herbivorous pests. A similar number of sequences of the library matched uncharacterised olive transcripts, suggesting that the olive response to *B. oleae* also involves novel or undescribed genes. The functional annotation of the library showed that a critical barrier for working with the olive is the dependence on models of biochemical pathways, gene ontology repertoires and genomic information that are primarily based on model species [32]. These results also demonstrated that the SSH approach was an appropriate strategy to study the olive response. Furthermore, although many cellular processes and pathways inherent to pest resistance are evolutionarily conserved in plants [30], the library analysis suggested that the plasticity of signal transduction networks and the variety of defence compounds may be particularly pronounced for olive. This assumption is conceivable, considering not only the difference between this tree and model plants but also the strong co-evolutionary relationship of the specialist *B. oleae* with olive fruits. We anticipate that the uncharacterized unigenes may represent a reservoir of candidate defence genes and that their study may allow further insights into the mechanism of olive defence.

The proteomic analysis identified 19 proteins as differentially expressed after insect attack. Two of these entries were also identified in our transcriptomic dataset. As a percentage, the matching rate is similar to other studies based on the proteomic and transcriptomic analyses of non-model tree species [33-35]. Furthermore, considering that the low correlation between sequences deriving from mRNA or protein analytical approaches is usually explained by post-transcriptional, translational and/or post-translational regulation processes [36,37], our data are suggestive of the importance and the possible magnitude of post-transcriptional events for gene regulation during pest defence [38]. Finally, this comparison also shows that transcriptomic and proteomic data are complementary in plants, as in other organisms [39-43].

As plants present a variety of strategies against insects [30], it is expected that several molecular processes are involved in the defence mechanisms against the olive fruit fly. Functional annotation indicated that *B. oleae* larval feeding significantly diminished the abundance of various proteins related to photosynthesis and altered the amount of those related to carbohydrate metabolism, which included a reduced expression of serine hydroxymethyltransferase, which is important for photorespiration in mitochondria [44], and of enzymes related to carbohydrate catabolic processes. The downregulation of genes coding for photosynthetic proteins or the decline in the photosynthetic rate in attacked plants has been observed for different herbivores that feed on leaves [45]. It is interesting that we observed a similar effect on sink organs, thus supporting the proposition that primary metabolites could also function as signals in pest defence pathways [46]. A stress-related promotion of drupe maturation should also be taken into consideration [47]. Transcriptomic analysis also demonstrated that unigenes responding to stress represent the largest functional class of upregulated olive genes during B. oleae attack. Under this condition, proteomics revealed increased accumulation of beta-glucosidase (as transcriptomics), major latex proteins, which have been already reported as defensive proteins against insects [48,49], phosphogluconolactonase and 6-phosphogluconate dehydrogenase. A possible defensive role of the latter enzymes has been suggested [50]. Taken together, the data indicated a metabolic shift towards defence during larval feeding [30]. Olive direct defence employs a variety of inducible factors, which include genes that are known to be important in the determination of the plant resistance to herbivorous pests, such as those coding for proteinase inhibitors or hydrolytic enzymes (e.g., chitinases and glucosidases); however, PR genes are also activated. Approximately one-third of the functionally annotated unigenes are homologues to genes first described to be involved in plant–pathogen interactions. Overlap in the signalling pathways regulating pathogen-plant and insect-plant interactions has been reported in several instances [51]. For the olive, such overlap is reasonable considering that *B. oleae* has long been known to be associated with different bacterial species [52,53].

The production of compounds involved in direct defence should be mainly dependent on a network that includes reactive oxygen species and phytohormone signalling. The results from the transcriptomic and proteomic analyses were consistent in showing a remarkable enrichment of genes and proteins involved in the regulation of the redox status (such as the metallothionein-like proteins, GST, catalase, thioredoxins, and aldo-keto reductase), thus indicating that ROS production should be a relevant component of induced olive defence. The reduction of some proteins involved ROS metabolism is indicative of the plant cell effort to maintain homeostasis under stress condition, preventing direct damage from the possible production of highly reactive cytotoxic compounds [54]. In the future, it will be interesting to ascertain if ROS production in drupes is also a consequence of the oxidative damage of membrane integrity due to lipid peroxidation. The functional classification indicated the presence of clones that are expected to be members of gene families involved in jasmonate signal transduction (e.g., lipoxygenase and the lipid transfer) or phenylpropanoid metabolism (i.e., the trans-cinnamate 4-hydroxylase and the Caffeoyl-o-methyltransferase) pathways, both of which produce compounds that, in plants, range from physical and chemical defence against biotic stressors to signal molecules involved in local and systemic signalling [55-57]. Overall, as was also reported for the interaction between the fungus *S. oleaginea* and olive [58], our data show an overlap of different pathways for the fruit fly response and denote that the olive responses to pathogens and herbivores should share a number of components at the signalling level [59].

Considering the unfeasibility of studying the olive-fruit fly interaction in controlled conditions, we recapitulated the expression analysis of twenty genes in drupes harvested in a different year. The real-time assay confirmed the differential expression of many but not all clones, as in other studies [32,60], implying that the extent of drupe responses to larval feeding may depend on the amount of damage inflicted, on environmental conditions at the time in which insects feed on plants, and on plant resource availability and allocation [11]. It is therefore relevant that, in replicate years and in two different cultivars, the most prominent gene activation was detected for trypsin inhibitors. Nonetheless, even though we could distinguish the transcripts of the two trypsin proteinase inhibitors in our qRT-PCR experiments, we cannot fully exclude the possibility that we monitored the activity of more than one transcript because serine inhibitors in plants belong to a large multigene family. In several plant species, proteinase inhibitors of the serine, cysteine and aspartic families are highly activated by larval feeding. Serine proteinases are the most relevant enzymes detected in the gut of Lepidoptera, Coleoptera, Hemiptera, Homoptera and Diptera [61]. However, bioassays using serine protease inhibitors against Diptera are more limited than in other insect orders. It has been shown that the gut proteolytic systems of larvae of the Mediterranean fruit fly (*C. capitata*) rely mostly on basic proteinases, with trypsin-like serine proteinases being the most important [62]. For these reasons, we argue that trypsin inhibitors should be a major element of drupe defence reaction. An in vivo assay against the olive fruit fly using purified inhibitors would clarify whether these molecules could be established as a novel insect control strategy, based on bio-compounds, on targeting the production of insect digestive enzymes by RNAi [63], on the selection of highly expressing olive genotypes, and on their combination.

Among the SSH-enriched cDNA clones, two sequences, coding for a chitinase and a PRp27-like protein, were selected for further functional characterisation. We isolated their full-length cDNA and studied their expression in response to biological (adult puncture and larval feeding of *B. oleae*) and physical (mechanical wounding) stress.

The Oe-Chi I protein has the two signature motifs of family 19 of the chitinases. Chitinases have long been considered a significant component of plant defence because of their direct action against chitin-containing pestiferous and pathogenic organisms. Furthermore, some of these enzymes are also involved in developmental processes or are associated with abiotic stress. The presence of a putative signal peptide and the gene expression analysis strongly support a role in drupe defence for Oe-Chi I. Specifically, taking into account the magnitude of gene activation, it is tempting to speculate that Oe-Chi I may not be exclusively involved in biological processes activated by “generic” mechanical damage but that this gene plays a specific role in the onset of the reaction against larval feeding. Although chitinases in plants are primarily associated with fungal resistance, there are various reports documenting their activation in plants following pest attack [64]. Interestingly, a chitinase was found to be specifically activated by the application of Colorado beetle regurgitant, and the gene had the highest expression level after a continuous infestation [65]. Further work, primarily directed towards the characterisation of the Oe-Chi I protein product, will be performed to understand its possible effect against phytophagous pests [66,67].

The Oe-PRp27 gene was named after its high similarity to the *Nicotiana tabacum* NtPRp27, which codes for a secreted protein belonging to the Pathogenesis-Related 17 (PR17) family [29]. Homologues of NtPRp27 have been found in a wide variety of flowering plants and because of their transcriptional activation in response to pathogen infection and various elicitors, these genes are classified as encoding PR proteins. However, the roles of the members of the PR17 family have not been fully elucidated, especially because these genes are activated by various forms of stress (drought, wounding, ABA, ethylene and MeJA) [68], and their constitutive expression in transgenic plants does not necessarily lead to an increased resistance to pathogens [29,69]. It has been proposed that PR17 members may act in defence responses with relation to either cell wall metabolism or signal transduction [29], which would be consistent with the rapid activation of Oe-PR27 after wounding. Furthermore, Oe-PR27 is induced by *B. oleae* feeding and punctures, implying an involvement in insect defence. This result is not completely unexpected, as NtPR27 is also induced by JA and ethylene [70], and recently, the accumulation of an NtPR-like protein was reported during bacterial infection of grape [71]. Although the mode of action and specificity of the PR17 family remain to be determined [68], our data support a possible broad function of members of this group in the early stages of defence, rather than as antibiotic components directly acting against invading pathogens.

## Conclusion

This study is the first investigation of the transcriptome and proteome of olive drupes attacked by *B. oleae*. The data from complementary approaches were useful to allow the identification of molecular players and to outline the biological functions, cellular processes and pathways associated with the drupe defence reaction. This work also revealed interesting genes that could have important roles in olive resistance and, eventually, be useful for the development of novel control strategies.

## Methods

### Suppression subtractive hybridization (SSH) library construction

Control and infested fruits of the olive (*Olea europaea* L. var. ‘europaea’) cultivar ‘Moraiolo’ were harvested (110 days after flowering) from plants growing in Montefalco (Perugia, Italy). The larvae were manually removed from the infested fruits and visually inspected under a binocular microscope, and tissues were stored at −80°C until molecular analysis. We found and analysed fruits with one larva. Biological replicates of the same variety were obtained from the Olive Cultivar Collection held by the ‘Centro di Ricerca per l’Olivicoltura e l’Industria Olearia’ in Collececco (Spoleto, Perugia, Italy). The fruit tissue surrounding the feeding tunnels of third instar larvae was used for library construction. Total RNA was isolated using the RNeasy Plant Mini Kit (Qiagen, Milano, Italy), and the contaminating genomic DNA was removed by a DNase I (Qiagen) treatment, according to the manufacturer’s instructions. PolyA + mRNA was isolated using Dynabeads Oligo (dT)_25_ (Invitrogen, Milano, Italy), and cDNA synthesis and subtracted library preparation were conducted with the PCR-select cDNA Subtraction Kit (Clontech, Milano, Italy), according to the manufacturer’s instructions. The tester and driver libraries were prepared from 4.0 μg of polyA + mRNA of infested and control undamaged fruits, respectively. The subtractive PCR experiments were conducted after 27 cycles of primary PCR and 11 cycles of secondary PCR with Advantage cDNA Polymerase mix (Clontech).

### SSH library analysis

Blunt-end PCR products were cloned in pCR-Blunt IITOPO plasmid vector (Blunt TOPO PCR Cloning Kit; Invitrogen) according to the manufacturer’s protocol. Following blue-white selection [72], the colonies were chosen at random and re-plated on selective medium for subsequent analysis. Plasmid DNA was isolated from an overnight culture in liquid selective medium using a standard alkaline lysis procedure [72]. The presence and size of the insert were verified by restriction digestion using Eco RI (Promega, Milano, Italy). Sanger sequencing was performed using the M13 forward and reverse primers with the BigDye Terminator v. 3.1 Ready Reaction Cycle Sequencing Kit (Applied Biosystems, Milano, Italy). After reaction clean-up using the BigDye XTerminator Purification Kit (Applied Biosystems), the fragments were resolved and analysed in an Applied Biosystems 3130 sequencer at the GenoPom Laboratory (Portici, Napoli, Italy). The vector backbone and adapter sequences were trimmed manually. The clones were filtered to eliminate sequences with low-quality reads, repeats (including low-complexity DNA sequences and highly repetitive sequences), low information content (such as runs of a single amino acid or a few amino acids, or runs of pyrimidines or purines), or sizes smaller than 66 bp. To remove redundancy for subsequent bioinformatic analysis, we assembled ESTs to obtain unigenes, using the CAP3 software with the default parameters [73]. The redundancy of the library was then calculated according to the following formula: [number of sequences – (total number of contiguous + total number of singletons)]/(total number of sequences)]*100.

Singletons and assembled DNA sequences (unigenes) were compared against non-redundant nucleotide and protein databases at the National Center for Biotechnological Information (http://www.ncbi.nlm.nih.gov) using BLAST programs [74]. First, comparative analysis was performed against non-redundant protein database with blastX. For clones showing low similarity, the comparison was conducted with blastn against the non-redundant nucleotide database, the nucleotide Expressed Sequence Tag database (dbEST) and, finally, the OleaESTdb (http://www.oleadb.it/). Potential open reading frames (ORFs) were searched using the Expasy translate tool [75]. As olive is a ‘non-model’ species, a low e-value threshold was used to determine whether a BLAST similarity allowed for functional annotation transfer to our unigenes. We retrieved the Gene Ontology (GO) terms associated with the Blast hits considering informative sequences with an e-value lower than 1e-06. The GO annotation was conducted using the Blast2GO software [76] at the default parameters, generating a ‘Plant’ GO-Slim mapping for the available annotations with a cut-off of two sequences. Conserved domains were searched in the predicted protein sequence database (http://www.ncbi.nlm.nih.gov/Structure/cdd/wrpsb.cgi) [77]. Subcellular localisation prediction was performed using the TargetP software (http://www.cbs.dtu.dk/services/TargetP) [78]. The iPSORT software was used to predict Signal Peptides in protein sequences [79].

### Real time PCR

For the transcriptional study of gene expression by real-time PCR, control and infested fruits of the olive cultivar ‘Moraiolo’ were harvested 110 days after flowering from plants growing in Montefalco (Perugia, Italy). One microgram of total RNA, isolated as described above, was treated with DNAse I (New England Biolabs, Milano, Italy) and reverse-transcribed with the RevertAid First Strand cDNA Synthesis Kit (Fermentas, Milano, Italy). The RNA quantity and quality were estimated spectrophotometrically (Biophotometer, Eppendorf, Milano, Italy). The PCR primers used in the qRT-PCR expression studies were designed using the Primer Express 2.0 software (Applied Biosystems). The primers and the sizes of the expected amplicons are indicated in the ` 5. The amplification of the cDNA coding for the Elongation Factor 1-α gene (Acc. Num. AM946404.1) served as a control for cDNA synthesis and PCR efficiency in the different samples. The sequences annealed by the two primers EF1-For and EF1-Rev are localised in two contiguous exons for the detection of possible contaminant DNA in the PCR amplifications. The real-time PCR experiments were performed using the ABI PRISM 7900HQ Sequence Detection System (Applied Biosystems, Foster City, CA, USA) as described [80]. Three independent amplifications were performed for each cDNA sample, and the reactions were performed for two biological replicates. The thermal cycling program started with a step of 2 min at 50°C and 10 min at 95°C, followed by 35 cycles consisting of 15 sec at 95°C followed by 1 min at 58°C. After each assay, a dissociation kinetics analysis was performed to check the specificity of the amplification products. The reaction products were also resolved on an agarose gel to verify the amplicon size. The relative quantification of the gene expression and its statistical test was conducted as previously described [81].

### Random amplification of cDNA ends (RACE) PCR

The recovery of full-length cDNA was performed by 5′ and 3′ rapid amplification of cDNA ends, using the 5′ RACE System for Rapid Amplification of cDNA Ends (Invitrogen) and the 3′ RACE System for Rapid Amplification of cDNA Ends (Invitrogen), according to the manufacturer’s instructions. Briefly, for the 5′ RACE, 5 μg of total RNA were reverse-transcribed using the SuperScript II enzyme (Invitrogen) and 0.5 μg of gene specific primer for 2 h, at 42°C. The sequences of the Oe-PRp27 and Oe-Chi I primers were 5′-GGAGCTTGACCATTTCCA-3′ and 5′-CCAATCCTGTCTTCCATTTG-3′, respectively. Second-strand cDNA synthesis was performed using the Abridged Universal Amplification Primer (Invitrogen) with the oligo 5′-TGATCTCCGTTTTCACATCACCA for Oe-PRp27 or 5′-GTTCGAACCTTTACCGCATTCAA for Oe-Chi I. The amplification conditions were one cycle of 94°C for 3 min; 40 cycles of 94°C for 30 sec, 55°C for 1 min and 72°C for 2 min; and a final step of 72°C for 10 min. For the 3′ RACE, the total RNA was retro-transcribed as above, using 0.5 μg of Adapter Primer (Invitrogen). The PCR amplification was conducted with either the Oe-PR27 gene-specific primer (5′- ATGTTTTGGGGCTTTCAATTTCC) or the Oe-Chi primer (5′- CCAACATCATCAATGGTGGA) along with the AUAP. The cycling conditions were as described above, except that the annealing temperature was 58°C. The PCR products were gel-purified using the QIAquick Gel Extraction Kit (Qiagen) and cloned into the pGEM-T Easy vector (Promega) according to the manufacturers’ instructions. The selection of recombinant colonies, plasmid DNA isolation and sequencing were performed as described above.

### Wounding treatment

Control and treated fruits of the olive cultivar ‘Leccino’ were treated 110 days after flowering. Drupes (of approximately 1.5 cm) were diagonally punctured (10 times) with a sterile steel needle without damaging the stone. Samples were harvested at time 0 (control), 24 and 48 h following treatment. Two pools of five drupes per plant were harvested from three separate branches. The drupes were manually destoned and frozen in liquid nitrogen until RNA isolation. The experiments were conducted in duplicate using drupes harvested from different trees.

### Protein extraction and 2-D electrophoresis

Drupes of three biological replicates of infested or control fruits from the cv. ‘Moraiolo’ were harvested at the same time, prepared and stored as described for the SSH library. Each biological replicate was independently subjected to a modified double protein extraction [82]. Briefly, 2.5 g of pulp were finely powdered in liquid nitrogen and suspended in 15 ml of ice-cold 10% trichloroacetic acid in acetone. After centrifugation at 10,000 g, for 5 min, at 4°C, the pellet was suspended in 10 ml of ice-cold 80% ammonium acetate in methanol and centrifuged as above. The pellet was suspended in 10 ml of ice-cold 80% acetone, centrifuged as above and resuspended in 7.5 ml of extraction buffer (30% sucrose, 2% SDS, 2% w/v β-mercaptoethanol, 1 mM PMSF, 1 mM Protease Inhibitor Cocktail (Sigma, Milano, Italy), 0.1 M Tris–HCl; pH 8.0). After addition of an equal volume of saturated phenol in 500 mM Tris–HCl, pH 7.5, the mixture was stirred for 10 min and then centrifuged at 10,000 g, for 15 min, at 4°C. The upper phenol phase was removed and extracted twice with the extraction buffer. Proteins were recovered from the phenol phase by addition of 5 vol of saturated ammonium acetate in methanol, overnight, at −20°C, and centrifuged at 10,000 g, for 30 min. Protein samples were then stored at −80°C.

Protein extracts were washed once with ice-cold methanol and three times with ice-cold acetone, dried under reduced pressure and dissolved in IEF buffer (9 M urea, 4% w/v CHAPS, 0.5% v/v Triton X-100, 20 mM DTT, 1% w/v carrier ampholytes pH 3–10, Bio-Rad, Hercules, CA, USA). Protein concentration was calculated by using the Bio-Rad protein assay, with BSA as a standard. IPG strips (17 cm, pH 5–8, ReadyStrip) (Bio-Rad) were rehydrated overnight with 300 μl of IEF buffer containing 400 μg of total proteins. Proteins were focused using a Protean IEF Cell (Bio-Rad) at 12°C as described [83]. After focusing, the proteins were reduced by incubating the IPG strips with 1% w/v DTT, for 15 min, and alkylated with 2.5% w/v iodoacetamide in 10 ml of equilibration buffer (6 M urea, 30% w/v glycerol, 2% w/v SDS, 50 mM Tris–HCl pH 8.8, and a dash of bromophenol blue) for 15 min. Electrophoresis in the second dimension was carried out on 12% polyacrylamide gels (180 x 240 x 1 mm) with the Protean apparatus (Bio-Rad), using electrophoresis buffer (1% w/v SDS, 1.92 M glycine, 25 mM Tris–HCl, pH 8.3), with 120 V applied for 12 h, until the dye front reached the bottom of the gel. Gels were stained with colloidal Coomassie G250. Each biological replicate from infested or control fruits was run in triplicate. Gel image acquisition and analysis was performed as described [83]. After normalization of the spot densities against the whole-gel densities, the percentage volume of each spot was averaged for nine different (three technical replicates of three biological samples) gels [24,35]. A two-fold change in normalized spot densities was considered indicative of a differentially synthesized protein (Student’s *t*-test).

### In gel digestion, mass spectrometry analysis and protein identification

Spots were excised from gels, triturated, in-gel reduced, S-alkylated and digested with trypsin, as previously reported [84]. Digests were subjected to desalting on ZipTipC18 (Millipore, Bedford, MA, USA), using 5% formic acid/50% acetonitrile as eluent, and analyzed by nanoLC-ESI-LIT-MS/MS with a LTQ XL mass spectrometer (Thermo, San Jose, CA, USA) equipped with Proxeon nanospray source connected to an Easy-nanoLC (Proxeon, Odense, Denmark) [85]. Peptide mixtures were separated on an Easy C18 column (10 × 0.075 mm, 3 μm) (Proxeon) by using a linear gradient from 5% to 50% of acetonitrile in 0.1% formic acid, over 60 min, at a flow rate of 300 nl/min. Spectra were acquired in the range m/z 400–2000. Acquisition was controlled by a data-dependent product ion scanning procedure over the three most abundant ions, enabling dynamic exclusion (repeat count 2 and exclusion duration 1 min). The mass isolation window and collision energy were set to *m/z* 3 and 35%, respectively.

MASCOT software package (Matrix Science, UK) was used to identify spots unambiguously from either an updated database containing all *O. europea* ESTs available over the WEB, or a plant non-redundant sequence database (NCBI nr 2009/05/03). Data were searched by using a mass tolerance value of 2 Da for precursor ion and 0.8 Da for MS/MS fragments, trypsin as proteolytic enzyme, a missed cleavages maximum value of 2 and Cys carbamidomethylation and Met oxidation as fixed and variable modification, respectively. Candidates with more than 2 assigned peptides with an individual MASCOT score >25, corresponding to *p* < 0.05 for a significant identification, were further evaluated by the comparison with their calculated mass and pI values, using the experimental values obtained from 2-DE.

## Abbreviations

EST, Expressed Sequence Tag; MS, Mass Spectrometry; ORF, Open Reading Frames; PR, Pathogenesis-Related; qRT-PCR, quantitative Real-Time PCR; RACE, Random Amplification of cDNA Ends; ROS, Reactive Oxygen Species; SSH, Suppression Subtractive Hybridisation.

## Competing interests

The authors declare that they have no competing interests.

## Authors’ contribution

GC designed experiments, analysed the data and wrote the paper, FA performed gene expression analysis, RACE-PCRs and GO analysis, MR and GR performed the proteomic work, PV constructed subtractive libraries, VC performed the experiments of the wounding assay and provided assistance in the GO analysis, MC was involved in the GO analysis, AG provided expertise in the classification of harvested material, LB provided plant material and participated in the study design, AS analysed the proteomic data, RR conceived and designed the study and reviewed the manuscript. All authors read and approved the manuscript.

## Supplementary Material

Additional file 1 **Candidate genes isolated from the SSH library**. (DOCX 19 kb) Click here for file

Additional file 2 BlastN similarity of the SSH clones to nucleotide sequences. (DOC 110 kb) Click here for file

Additional file 3 **The real-time PCR analysis of the expression level of the Trypsin inhibitor II transcripts in the olive cultivar ‘Leccino’.** The graph displays the relative quantification of the gene expression (RQ) in drupes with feeding tunnels (infested) relative to uninfested drupes (control), set as the calibrator. Asterisks indicate significant difference compared to control (*p* < 0.01). (DOCX 12 kb) Click here for file

Additional file 4 **Proteins with changed expression levels in O*. europaea*fruits after damage by*Bactrocera oleae*.** The spot number, protein name, gene/EST number according to the NCBI database, protein entry in the NCBI database with the highest BLAST score, experimental pI/Mr values, peptide number/sequence coverage (%), Mascot score, identification method, organism, fold change for infested vs. control plants and GO classification are listed. All of the spots were identified by tandem MS. (DOCX 17 kb) Click here for file

Additional file 5 Primers used for the expression study and their main features. (DOCX 15 kb) Click here for file
