# Supplementary material for: Molecular interactions between the olive and the fruit fly Bactrocera oleae
Source: BMC Plant Biol. 2012 Jun 13;12:86. doi: 10.1186/1471-2229-12-86 (PMC3733423; doi:10.1186/1471-2229-12-86)
Supplement: Additional file 1 — Candidate genes isolated from the SSH library. (DOCX 19 kb) [file 1471-2229-12-86-S1.docx]

**Additional file1. Candidate genes isolated from the SSH library.**

| ID | Acc. Num.* | Size (bp) | Best Hit using tBlastX [Species]; Genebank Accession number | e-Value | Local Similarity |
| --- | --- | --- | --- | --- | --- |
| 2 | JN696113 | 269 | Extracellular chitinase, class I [Vitis vinifera]; XP_002269972.1 | 7.55E-25 | 96% |
| 6-F | JQ711527 | 304 | RNA export [Nicotiana benthamiana]; ABG29731.1 | 8.86E-26 | 93% |
| 7-F | JQ711518 | 480 | Predicted protein [Populus trichocarpa]; EEE92758.1 | 2.82E-32 | 82% |
| 8 | JQ711506 | 354 | Beta-amylase [Nicotiana langsdorffii x Nicotiana sanderae]; AAY89374.1 | 3.06E-31 | 81% |
| 11-L | JQ711507 | 233 | Caffeoyl- o-methyltransferase [Coffea canephora]; ABO77959.1 | 3.39E-09 | 96% |
| 16 | JQ711504 | 322 | 50S ribosomal protein L33 [Ricinus communis]; EEF45456.1 | 4.77E-11 | 82% |
| 31-L | JQ711515 | 530 | GDP-fucose protein-o-fucosyltransferase 2 [Solanum tuberosum]; CAE30293.1 | 1.98E-40 | 96% |
| 35 | JQ711510 | 428 | DNA binding protein [Cucumis melo]; ADN34037.1 | 2.68E-22 | 79% |
| 38 | JQ711531 | 355 | Thioredoxin H-type [Hevea brasiliensis]; AAD33596.1 | 8.59E-37 | 87% |
| 51-F | JQ711535 | 286 | Uncharacterized protein [Glycine max]; ACU13472.1 | 3.93E-21 | 98% |
| 52-F | JQ711522 | 201 | ORF able to induce HR lesions [Nicotiana tabacum]; AAC49976.1 | 2.51E-12 | 86% |
| 52-L | JQ711520 | 215 | Metallothionein-like protein [Pimpinella brachycarpa]; AAC62510.1 | 2.35E-10 | 80% |
| 59-P | JQ711516 | 265 | Glutathione S-transferase [Hyoscyamus muticus]; P46423.1 | 2.87E-21 | 85% |
| 71-N | JQ711534 | 370 | TSL-kinase interacting protein 1-like [Glycine max] XP_003519867.1 | 6.02E-15 | 71% |
| 73-N | JQ711511 | 295 | EamA-like transporter family protein [Arabidopsis thaliana]; AEE86010.1 | 3.90E-13 | 90% |
| 74-N | JQ711525 | 309 | Perakine reductase [Rauvolfia serpentina]; AAX11684.1 | 3.46E-14 | 81% |
| 75-L | JQ711519 | 526 | Seed imbibitions protein 1 [Vitis vinifera]; ACD39775.1 | 1.06E-46 | 75% |
| 76-N | JQ711514 | 512 | inducible lysosomal thiol reductase isoform 2 [Vitis vinifera]; XP_002275283 | 4.02e-48 | 62% |
| 82-L | JQ711502 | 629 | Proteasome alpha 6 subunit [Nicotiana benthamiana]; AAN07899.1 | 7.26E-21 | 73% |
| 87-N | JQ429795 | 232 | Late embryogenesis abundant protein [Nicotiana tabacum]; AAC06242.1 | 4.62E-06 | 77% |
| 88 | JQ711512 | 321 | Elicitor-inducible protein EIG-J7 [Capsicum annuum] | 1.14E-22 | 55% |
| 93 | JQ429797 | 249 | Trypsin chymotrypsin inhibitor [Lens culinaris]; CAR47883.1 | 1.10E-36 | 100% |
| 95 | JQ711508 | 309 | cellulose synthase-like protein [Vitis vinifera]; XP_002518853.1 | 2.01E-25 | 53% |
| 98-L | JQ711538 | 519 | Xylose isomerase [Ricinus communis]; EEF29973.1 | 2.05E-31 | 97% |
| 99-N | JQ429796 | 233 | Proteinase inhibitor type-2 [Solanum lycopersicum]; CAA64416.1 | 1.14E-12 | 58% |
| 102 | JQ711503 | 450 | 40s ribosomal protein s17 [Solanum lycopersicum]; AAD50774.1 | 8.52E-61 | 94% |
| 103-L | JQ711517 | 310 | H/ACA ribonucleoprotein complex subunit [Ricinus communis]; XP_002512015.1 | 4.78E-40 | 94% |
| 109-N | JQ711533 | 336 | Transducin family protein [Arabidopsis lyrata]; EFH62484.1 | 4.52E-22 | 92% |
| 119 | ABS72010.1 | 200 | Catalase [Olea europaea]; ABS72010.1 | 5.60E-23 | 100% |
| 143-2 | JQ711505 | 216 | Adipocyte plasma membrane-associated protein, putative [Ricinus communis]; EEF46261.1 | 1.01E-04 | 58% |
| 148 | JQ711526 | 386 | Aquaporin PIP2 [Vitis vinifera]; ABN14353.1 | 3.60E-19 | 95% |
| 237 | JQ711513 | 355 | Enolase [Solanum lycopersicum]; CAA41115.1 | 4.00E-18 | 97% |
| 301-P | EU513351 | 229 | Lipoxygenase [Arabidopsis thaliana]; CAC19365.1 | 7.00E-13 | 72% |
| C2 | JN696114 | 647 | NtPRp27 [Nicotiana tabacum]; BAA81904.1 | 1.07E-78 | 95% |
| C4 | JQ711509 | 229 | Disease resistance response protein 206 [Zea mays]; NP_001149569.1 | 9.94E-09 | 73% |
| C6 | JQ711524 | 465 | Pathogenesis-related protein 10.5 [Vitis vinifera]; CBJ49377.1 | 1.17E-33 | 83% |
| C7 | ABS72007.1 | 445 | Putative apyrase [Olea europaea]; ABS72007.1 | 5.94E-70 | 98% |
| C9 | JQ711523 | 411 | Pathogenesis-related thaumatin-like protein [Coffea arabica]; ABW76502.1 | 9.75E-25 | 75% |
| C10 | JQ711521 | 427 | Non-specific lipid-transfer protein type 2 [Nicotiana tabacum]; BAJ25798.1 | 2.51E-28 | 85% |
| C12 | JQ711536 | 670 | Unknown protein [Ricinus communis]; EEF48224.1 | 5.65E-69 | 81% |
| C13 | JQ429798 | 279 | Ubiquitin-conjugating enzyme E2 [Ricinus communis]; XP_002523377.1 | 7.64E-46 | 100% |
| C15 | JQ711537 | 320 | Universal stress protein family protein [Hordeum vulgare]; ADB54810.1 | 4.94E-24 | 82% |
| C17 | AAL93619.1 | 940 | Beta-glucosidase [Olea europaea]; AAL93619.1 | 1.08E-56 | 100% |
| C25 | JQ711530 | 606 | Thioredoxin H-type 1 [Nicotiana benthamiana]; ACV52592.1 | 2.07E-46 | 90% |
| C26 | JQ711529 | 349 | Signal peptidase complex subunit 1 [Oryza sativa]; ABA98316.2 | 4.95E-08 | 88% |
| C29 | JQ711528 | 372 | Serine carboxypeptidase [Ricinus communis]; EEF40335.1 | 1.22E-14 | 72% |
| C32 | JQ711532 | 553 | Trans-cinnamate 4-hydroxylase [Populus tremuloides]; ABF69101.1 | 5.56E-39 | 99% |

* Note: for clone 2 and C2 it is reported the accession number of the rescued full length sequences; for clone 119, 301-P, C7, C17 the accession number of the corresponding available sequence at NCBI.
