# Supplementary material for: Molecular interactions between the olive and the fruit fly Bactrocera oleae
Source: BMC Plant Biol. 2012 Jun 13;12:86. doi: 10.1186/1471-2229-12-86 (PMC3733423; doi:10.1186/1471-2229-12-86)
Supplement: Additional file 2 — BlastN similarity of the SSH clones to nucleotide sequences. (DOC 110 kb) [file 1471-2229-12-86-S2.docx]

**Additional file 2: BlastN similarity of the SSH clones to nucleotide sequences.**

| **ID** | **dbES Id** | **Acc. Numb.** | **Size (bp)** | **Best hit [species]; accession number** | **Search set for BlastN^a^** | **e-Value** | **Local similarity** |
| --- | --- | --- | --- | --- | --- | --- | --- |
| 3F | 76276260 | JK784468 | 276 | Cu/Zn super-oxide dismutase (Ole e 5 allergen) [Olea europaea]; AJ428575.2 | nr | 2,09E-11 | 96% |
| 5F | 76276261 | JK784469 | 259 | Ubiquinol-cytochrome-c reductase like protein [Arabidopsis thaliana]; AK226409.1 | nr | 5,24E-11 | 87% |
| 12 | 76276262 | JK784470 | 131 | C2H2 zinc finger protein [Triticum aestivum]; EF592555.1 | nr | 1,44E-07 | 86% |
| 15 | 76276263 | JK784471 | 319 | Clone: LEFL1026DG03, HTC in leaf [Solanum lycopersicum]; AK321625.1 | nr | 6,19E-05 | 75% |
| 27 | 76276264 | JK784472 | 176 | Protein UP16 [Olea europaea]; EF506551.1 | nr | 5,96E-65 | 94% |
| 27F | 76276265 | JK784473 | 318 | Hypothetical protein [Vitis vinifera]; XM_002268025.1 | nr | 5,06E-06 | 90% |
| 36 | 76276266 | JK784474 | 381 | Acidic proline-rich protein PRP25 precursor-like [Arachis hypogaea]; DQ296039.1 | nr | 2,16E-05 | 80% |
| 36-L | 76276267 | JK784475 | 242 | Predicted protein [Populus trichocarpa]; XM_002315115.1 | nr | 1,14E-31 | 96% |
| 56F | 76276270 | JK784478 | 242 | Clone MGBa [Mimulus guttatus]; AC182573.2 | nr | 4,84E-11 | 79% |
| 73 | 76276271 | JK784479 | 516 | St1-bp8 microsatellite sequence [Probarbus jullieni]; GQ130220.1 | nr | 1,05E-04 | 82% |
| 73-L | 76276272 | JK784480 | 224 | Similar to AT4g27960 mRNA [Vitis vinifera]; XM_002274238.1 | nr | 1,19E-05 | 97% |
| 75 | 76276273 | JK784481 | 328 | Pb01 cortical actin cytoskeleton protein [Paracoccidioides brasiliensis]; XM_002796459.1 | nr | 7,78E-04 | 88% |
| 79N | 76276274 | JK784482 | 176 | Predicted protein [Populus trichocarpa]; XM_002297921.1 | nr | 1,71E-08 | 85% |
| 114-L | 76276275 | JK784483 | 171 | Plant lipid transfer protein [Olea europaea]; EF506504.1 | nr | 4,73E-09 | 88% |
| 118-L | 76276276 | JK784484 | 138 | Diphosphomevalonate decarboxylase [Ricinus communis]; XM_002521126.1 | nr | 2,29E-05 | 82% |
| 257 | 76276277 | JK784485 | 243 | Cytosolic ascorbate peroxidase [Solanum tuberosum]; AY742959.1 | nr | 3,07E-07 | 88% |
| C1 | 76276278 | JK784486 | 424 | Bax inhibitor-1 [Nicotiana tabacum]; AF390556.1 | nr | 5,01E-14 | 93% |
| C3 | 76276279 | JK784487 | 232 | Aquaporin PIP2.1 [Ricinus communis]; XM_002530234.1 | nr | 5,62E-10 | 89% |
| C5 | 76276280 | JK784488 | 331 | Metallothionein 2 [Plantago major]; AJ843994.1 | nr | 1,33E-13 | 78% |
| C11 | 76276281 | JK784489 | 226 | Pathogenesis-related protein (PR-10) [Rehmannia glutinosa]; EU526395.1 | nr | 8,08E-08 | 74% |
| C21 | 76276284 | JK784492 | 336 | EST from severe drought-stressed opposite wood [Populus tremula x Populus alba]; CU233079.1 | nr | 1,27E-07 | 83% |
| C23A | 76276285 | JK784493 | 164 | Enoyl ACP reductase [Olea europaea]; AY083164.1 | nr | 6,95E-20 | 95% |
| C23B | 76276286 | JK784494 | 272 | Enoyl ACP reductase [Olea europaea]; AY083164.1 | nr | 2,06E-19 | 95% |
| C27 | 76276287 | JK784495 | 361 | Glyceraldehyde-3-phosphate dehydrogenase [Nicotiana tabacum]; AJ133422.1 | nr | 1,46E-13 | 90% |
| C28 | 76276288 | JK784496 | 321 | Hypothetical protein [Vitis vinifera]; XM_002264720.1 | nr | 5,12E-06 | 78% |
| C30 | 76276289 | JK784497 | 400 | Hypothetical protein [Vitis vinifera]; XM_002275090.1 | nr | 8,49E-11 | 82% |
| 31 | 76276290 | JK784498 | 167 | Pathogenesis-related transcriptional factor and ERF domain containing protein [Oryza sativa ]; NM_001065752.1 | EST | 3,07E-04 | 55% |
| 233 | 76276291 | JK784499 | 252 | Glucose acyltransferase (pldp15) [Solanum berthaultii]; AF006080.1 | EST | 5,26E-05 | 60% |
| 55FA | 76276268 | JK784476 | 218 | Unknown transcript [Olea europaea]; [OLEEUCl087483:Contig1](http://140.164.45.140/oleaestdb/clusterview.php?clid=OLEEUCl087483) | Olea db | 7,00E-40 | 95% |
| C20A | 76276282 | JK784490 | 180 | Metallothionein-like protein [Olea europaea]; [OLEEUCl011053:Contig4](http://140.164.45.140/oleaestdb/clusterview.php?clid=OLEEUCl011053) | Olea db | 7,00E-69 | 100% |
| 55FB | 76276269 | JK784477 | 270 | Putative syntaxin-24 [Olea europaea]; [OLEEUCl067506:Contig1](http://140.164.45.140/oleaestdb/clusterview.php?clid=OLEEUCl067506) | Olea db | 2,00E-39 | 98% |
| 2F | 76276292 | JK784500 | 219 | Unknown transcript [Olea europaea]; OLEEUCl052033:C1 | Olea db | 9,00E-75 | 96% |
| 33 | 76276293 | JK784501 | 186 | Beta-1,3-glucanase [Olea europaea]; E8NTSAO02DV56C | Olea db | 1,00E-08 | 84% |
| 46-L | 76276294 | JK784502 | 239 | Unknown transcript [Olea europaea]; OLEEUCl080025:Contig1 | Olea db | 1,00E-80 | 100% |
| 53F | 76276295 | JK784503 | 193 | Ethylene-responsive transcription factor (ERF) [Olea europaea]; OLEEUCl001226:Contig3 | Olea db | 1,00E-100 | 98% |
| 71 | 76276296 | JK784504 | 326 | Unknown transcript [Olea europaea]; OLEEUCl066120:Contig1 | Olea db | 6,00E-12 | 92% |
| 78 | 76276297 | JK784505 | 100 | 60S ribosomal protein L37 [Olea europaea]; OLEEUCl009088:Contig1 | Olea db | 9,00E-42 | 98% |
| 79 | 76276298 | JK784506 | 206 | Proteinase-like cysteine protease [Olea europaea]; OLEEUCl073464:Contig1 | Olea db | 3,00E-59 | 99% |
| 97-N | 76276299 | JK784507 | 176 | S-adenosylmethionine synthase 1 [Olea europaea]; OLEEUCl081096:Contig1 | Olea db | 3,00E-49 | 98% |
| 100 | 76276300 | JK784508 | 264 | Axial regulator YABBY 5 [Olea europaea]; OLEEUCl018960:Contig4 | Olea db | 2,00E-08 | 89% |
| 121-L | 76276301 | JK784509 | 205 | Unknown transcript [Olea europaea]; OLEEUCl010627:Contig1 | Olea db | 1,00E-58 | 100% |
| 122 | 76276302 | JK784510 | 142 | Unknown transcript [Olea europaea]; OLEEUCl033208:Contig1 | Olea db | 5,00E-75 | 100% |
| 128 | 76276303 | JK784511 | 86 | Cytochrome P450 [Olea europaea]; E8NTSAO03F5L17 | Olea db | 2,00E-26 | 93% |
| 129 | 76276304 | JK784512 | 157 | Unknown transcript [Olea europaea]; OLEEUCl026805:Contig1 | Olea db | 2,00E-31 | 97% |
| 130 | 76276305 | JK784513 | 69 | Unknown transcript [Olea europaea]; OLEEUCl043704:Contig1 | Olea db | 5,00E-18 | 92% |
| 139 | 76276306 | JK784514 | 162 | Osmotin-like protein [Olea europaea]; E8NTSAO03GO5ZY | Olea db | 7,00E-47 | 97% |
| 144 | 76276307 | JK784515 | 190 | Unknown transcript [Olea europaea]; E8NTSAO03GGFKB | Olea db | 4,00E-15 | 94% |
| 230 | 76276308 | JK784516 | 155 | Unknown transcript [Olea europaea]; E8NTSAO03GW7CJ | Olea db | 2,00E-75 | 98% |
| 252 | 76276309 | JK784517 | 145 | Malate dehydrogenase [Olea europaea]; OLEEUCl017582:Contig2 | Olea db | 2,00E-77 | 100% |
| C14 | 76276310 | JK784518 | 243 | Unknown transcript [Olea europaea]; OLEEUCl021129:Contig1 | Olea db | 9,00E-29 | 100% |
| C18 | 76276311 | JK784519 | 574 | MLP-like protein [Olea europaea]; OLEEUCl009668:Contig5 | Olea db | 1,00E-177 | 97% |
| C22 | 76276312 | JK784520 | 290 | Unknown transcript [Olea europaea]; OLEEUCl067242:Contig1 | Olea db | 1,00E-133 | 99% |
| C24 | 76276313 | JK784521 | 326 | Methyl esterase [Olea europaea]; OLEEUCl004759:Contig1 | Olea db | 1,00E-175 | 99% |
| C31 | 76276314 | JK784522 | 218 | Actin [Olea europaea]; OLEEUCl086181:Contig1 | Olea db | 5,00E-30 | 93% |
| C20B | 76276283 | JK784491 | 263 | - | - | - | - |

* nr: non-redundant nucleotide collection at NCBI; EST: Expressed Sequence Tags collection at NCBI; Olea db: tentative consensus and singletons at the Olea EST db.
