# Supplementary material for: Molecular interactions between the olive and the fruit fly Bactrocera oleae
Source: BMC Plant Biol. 2012 Jun 13;12:86. doi: 10.1186/1471-2229-12-86 (PMC3733423; doi:10.1186/1471-2229-12-86)
Supplement: Additional file 3 — The real-time PCR analysis of the expression level of the Trypsin inhibitor II transcripts in the olive cultivar ‘Leccino’. The graph displays the relative quantification of the gene expression (RQ) in drupes with feeding tunnels (infested) relative to uninfested drupes (control), set as the calibrator. Asterisks indicate significant difference compared to control (p < 0.01). (DOCX 12 kb) [file 1471-2229-12-86-S3.docx]

**Additonal file 3**

**

The real-time PCR analysis of the expression level of the Trypsin inhibitor II transcripts in the olive cultivar ‘Leccino’. The graph displays the relative quantification of the gene expression (RQ) in drupes with feeding tunnels (infested) relative to uninfested drupes (control), set as the calibrator. Asterisks indicate significant difference compared to control (*p*<0.01).
