# Supplementary material for: Molecular interactions between the olive and the fruit fly Bactrocera oleae
Source: BMC Plant Biol. 2012 Jun 13;12:86. doi: 10.1186/1471-2229-12-86 (PMC3733423; doi:10.1186/1471-2229-12-86)
Supplement: Additional file 4 — Proteins with changed expression levels in O. europaeafruits after damage byBactrocera oleae. The spot number, protein name, gene/EST number according to the NCBI database, protein entry in the NCBI database with the highest BLAST score, experimental pI/Mr values, peptide number/sequence coverage (%), Mascot score, identification method, organism, fold change for infested vs. control plants and GO classification are listed. All of the spots were identified by tandem MS. (DOCX 17 kb) [file 1471-2229-12-86-S4.docx]

**Additional file 4. Proteins with changed expression levels in O*. europaea* fruits after damage by *Bactrocera oleae*.**

The spot number, protein name, gene/EST number according to the NCBI database, protein entry in the NCBI database with the highest BLAST score, experimental pI/Mr values, peptide number/sequence coverage (%), Mascot score, identification method, organism, fold change for infested vs. control plants and GO classification are listed. All of the spots were identified by tandem MS.

| Spot | Protein Name | Protein Gi/Est Code | NCBI Blast result | Experimental  pI/Mr (kDa) | Peptides/ Sequence cov. (%) | Mascot Score | Organism | Fold change (larvae/control) | Function |
| --- | --- | --- | --- | --- | --- | --- | --- | --- | --- |
| 1 | 6-Phosphogluconolactonase | OLEEUCl015012 | 255554349  (R. communis) | 4.77/24.7 | 23/56 | 1313 | O. europaea | 4.8 | Carbohydrate metabolism; recovery from redox shunt/NADPH synthesis |
| 2 | 6-Phosphogluconolactonase | OLEEUCl015012 | 255554349  (R. communis) | 4.83/25.4 | 12/37 | 561 | O. europaea | 0.2 | Carbohydrate metabolism; recovery from redox shunt/NADPH synthesis |
| 3 | Superoxide dismutase | OLEEUCl006293 | 38228697  (F. sylvatica) | 5.41/11.2 | 4/15 | 246 | O. europaea | 0.2 | Redox process |
| 4 | Cyclophilin | 145049729 |  | 6.50/14.2 | 2/3 | 172 | I. batatas | 0.1 | Protein folding |
| 5 | Major latex protein | gi\|242394555 | 255581166  (R. communis) | 4.83/13.8 | 13/37 | 636 | O. europaea | 2.4 | Defense response/toxic metabolite delivery |
| 6 | Thioredoxin-dependent peroxidase | gi\|242394658 | 52851172  (P. major) | 4.97/17.2 | 6/23 | 301 | O. europaea | 0.4 | Redox process |
| 7 | Disease resistance response protein 206 | OLEEUCl007459 | 226498948  (Z. mays) | 5.65/20.8 | 2/9 | 122 | O. europaea | 0.3 | Defense response |
| 8 | Beta-glucosidase | gi\|25989473 | 25989474  (O. europaea) | 5.51/66.0 | 21/33 | 1207 | O. europaea | 37.1 | Carbohydrate metabolism |
| 9 | Enolase | OLEEUCl012406 | 119354 (S. lycopersicum) | 5.49/51.4 | 21/50 | 1371 | O. europaea | 7.2 | Carbohydrate metabolism |
| 10 | 6-Phosphogluconate dehydrogenase | OLEEUCl019391 | 255537671  (R. communis) | 5.51/48.0 | 4/22 | 174 | O. europaea | 12.2 | Carbohydrate metabolism; recovery from redox shunt/NADPH synthesis |
| 11 | Phenylcoumaran benzylic ether reductase | OLEEUCl002705 | 213385143  (N. tabacum) | 5.50/36.2 | 14/31 | 635 | O. europaea | 2.2 | Metabolite transport |
| 12 | Aldo/keto reductase, putative | OLEEUCl006795 | 255542314  (R. communis) | 6.15/41.8 | 13/24 | 720 | O. europaea | 0.4 | Redox process |
|  | Fructose-bisphosphate aldolase-like protein | gi\|242394622 | 83283995  (S. tuberosum) |  | 6/26 | 510 | O. europaea |  | Carbohydrate metabolism |
|  | Glyceraldehyde-3-phosphate dehydrogenase | OLEEUCl019949 | 4539543  (N. tabacum) |  | 10/31 | 495 | O. europaea |  | Carbohydrate metabolism |
| 13 | Fructose-bisphosphate aldolase-like protein | gi\|242394622 | 83283995  (S. tuberosum) | 6.21/42.7 | 9/39 | 670 | O. europaea | 0.3 | Carbohydrate metabolism |
|  | Aldo/keto reductase, putative | OLEEUCl006795 | 255542314  (R. communis) |  | 12/28 | 623 | O. europaea |  | Redox process |
|  | Glyceraldehyde-3-phosphate dehydrogenase | OLEEUCl004899 | 4539543  (N. tabacum) |  | 8/18 | 455 | O. europaea |  | Carbohydrate metabolism |
| 14 | Fructose-bisphosphate aldolase-like protein | OLEEUCl016308 | 78191410  (S. tuberosum) | 6.27/42.8 | 15/40 | 928 | O. europaea | 0.2 | Carbohydrate metabolism |
|  | Glyceraldehyde-3-phosphate dehydrogenase | OLEEUCl004899 | 4539543  (N. tabacum) |  | 4/9 | 245 | O. europaea |  | Carbohydrate metabolism |
| 15 | Short chain alcohol dehydrogenase | OLEEUCl019680 | 255565739  (R. communis) | 6.54/26.7 | 3/10 | 140 | O. europaea | 0.3 | Redox process |
| 16 | Short chain alcohol dehydrogenase | OLEEUCl019680 | 255565739  (R. communis) | 6.34/25.6 | 7/35 | 495 | O. europaea | 0.1 | Redox process |
| 17 | Not identified |  |  | 6.44/32.2 |  |  |  | 0.2 |  |
| 18 | Methylthioadenosine nucleotidase | OLEEUCl007515 | 224119752  (P. trichocarpa) | 6.42/36.2 | 5/6 | 234 | O. europaea | 0.2 | Nucleotide/ethylene metabolism |
| 19 | Not identified |  |  | 6.65/34.2 |  |  |  | 0.2 |  |
| 20 | Not identified |  |  | 6.66/32.4 |  |  |  | 0.2 |  |
| 21 | Short chain alcohol dehydrogenase | OLEEUCl019680 | 255565739  (R. communis) | 6.28/29.7 | 4/16 | 185 | O. europaea | 0.1 | Redox process |
| 22 | Formate dehydrogenase, mitochondrial | OLEEUCl045778 | 26454627  (S. tuberosum) | 6.27/46.2 | 10/21 | 409 | O. europaea | 0.3 | Redox process; recovery from redox shunt/NADPH synthesis |
| 23 | NADP-dependent isocytrate dehydrogenase | 147857569 |  | 6.25/47.3 | 3/8 | 143 | V. vinifera | 0.2 | Redox process; glyoxylate cycle |
| 24 | Serine hydroxymethyltransferase | OLEEUCl009950 | 255563608  (R. communis) | 6.74/53.6 | 3/8 | 208 | O. europaea | 0.2 | Amino acid metabolism |
| 25 | Major latex-like protein | OLEEUCl009668 | 197725474  (P. ginseng) | 5.64/12.8 | 4/19 | 218 | O. europaea | 2.2 | Defense response/toxic metabolite delivery |
| 26 | Fructose-bisphosphate aldolase-like protein | gi\|242394622 | 83283995  (S. tuberosum) | 6.49/45.2 | 11/39 | 839 | O. europaea | 0.3 | Carbohydrate metabolism |
|  | Glyceraldehyde-3-phosphate dehydrogenase | OLEEUCl004899 | 4539543  (N. tabacum) |  | 10/21 | 485 | O. europaea |  | Carbohydrate metabolism |
