# Supplementary material for: Molecular interactions between the olive and the fruit fly Bactrocera oleae
Source: BMC Plant Biol. 2012 Jun 13;12:86. doi: 10.1186/1471-2229-12-86 (PMC3733423; doi:10.1186/1471-2229-12-86)
Supplement: Additional file 5 — Primers used for the expression study and their main features. (DOCX 15 kb) [file 1471-2229-12-86-S5.docx]

Supplementary table 5. Primers used for the expression study and their main features.

% GC: percentage of G and C; AL: amplicon length (bp); Ta: annealing temperature (°C).

| Primer | Sequence (5’ to 3’) | Gene (Accession number) | Tm | % GC | AL | Ta |
| --- | --- | --- | --- | --- | --- | --- |
| AQ-For | TCTCGGGCCCTTGTTTTAGA | Aquaporin (JQ711526) | 58 | 50 | 91 | 56 |
| AQ-Rev | AAAGAGAGGCCAGCAACCG |  | 59 | 58 |  |  |
| Beta-GLU-For | TTGCCAAAACGTTCAGCTGT | Beta-glucosidase (AAL93619.1) | 58 | 45 | 110 | 58 |
| Beta-GLU-Rev | GCCTCTAAGCCTTTTACGACGAT |  | 59 | 48 |  |  |
| C4H-For | CGGCATTACTTTGGGACGTTT | Cinnamate 4 hydroxylase (JQ711532) | 60 | 48 | 101 | 59 |
| C4H-Rev | GCAGACTGAATTGGCCACCT |  | 59 | 55 |  |  |
| Carbox For | GTGTCGCAAATGTGAGATGG | Serine-carboxypeptidase-like protein (JQ711528) | 60 | 50 | 160 | 60 |
| Carbox Rev | GATCATGACATGGCTGCACT |  | 59 | 50 |  |  |
| CAT-For | CCGAAAATGGGTAGATGCTTTATC | Catalase (ABS72010.1) | 59 | 42 | 110 | 58 |
| CAT-Rev | GAGGCGAGCTTCTGACCAAG |  | 59 | 60 |  |  |
| CHIT II-For | TCGACTCCATGAACATCATCG | Chitinase I (JN696113) | 58 | 48 | 111 | 58 |
| CHIT II-Rev | GAGCCTGTTCGGCACAGGT |  | 60 | 63 |  |  |
| DisRes For | GCCATTCGGAAAACACCAGT | Disease resistance protein (JQ711509) | 62 | 50 | 160 | 60 |
| DisRes Rev | CTTTCAGCGGAAGCGTAATC |  | 59 | 50 |  |  |
| EF1-For | TGCACAGTTATTGATGCTCCA | Elongation factor -1α (AM946404.1) | 59 | 60 | 400 | 58 |
| EF1-Rev | GGGCTCCTGAATCTGGTCAA |  | 58 | 58 |  |  |
| Ethylen For | TCATGTGCGTTGTTGATGGT | Ethylene Responsive Protein (JZ331728) | 61 | 45 | 161 | 60 |
| Ethylen Rev | TGTCTTTTAACATTCACACAGA |  | 60 | 50 |  |  |
| GST-For | CTTTTCGATGAACGCCCTCA | Glutathione-S-transferase (JQ711516) | 60 | 50 | 91 | 59 |
| GST-Rev | AACGCTTCGTCATTGCAACA |  | 59 | 45 |  |  |
| LOX-For | CGGGTTGGACCAGTGAATGT | Lipoxygenase (EU513351) | 59 | 55 | 90 | 60 |
| LOX-Rev | TTGACACACTGTTGGGAATTCC |  | 58 | 45 |  |  |
| Metal-1 For | CCATAGCTACTTTGAAGGGCTTA | Metallothionein type 1 (JQ711520) | 59 | 43 | 170 | 60 |
| Metal-1 Rev | GTGGTCGCACCAAAGAAGAT |  | 60 | 50 |  |  |
| PR10-For | CAGGAGAAGCACCGTGACAA | PR 10 (JQ711524) | 58 | 74 | 100 | 58 |
| PR10-Rev | TCTGAAGATCGAGAATGCTTTCC |  | 59 | 54 |  |  |
| PR27-For | CCCAGAAGATCAACGAAGTAGGTG | PRp27 (JN696114) | 60 | 50 | 110 | 58 |
| PR27-Rev | GACCGCACGATTCTTGGATT |  | 59 | 50 |  |  |
| SOD- For | TGGTGGAAGAGTTGCTTGTGG | Superoxide dismutase (JK784468) | 60 | 52 | 110 | 60 |
| SOD- Rev | TGATGACCGAGTTTGTACACAGTG |  | 59 | 46 |  |  |
| TCI-For | TGTTGTGATACGTGCCTTTGC | Trypsin/chymotrypsin inhibitor (JQ429797) | 58 | 48 | 91 | 58 |
| TCI-Rev | CATTTGTCGCATGCAGAGTGA |  | 59 | 48 |  |  |
| TPI II For | TGTGCAGCTTGGATGAAGCT | Tripsin protease inhibitor II (JQ429796) | 58 | 56 | 112 | 60 |
| TPI II Rev | CAACGTGAACTATGCTGAAGCC |  | 59 | 56 |  |  |
| Transd For | GCTTGGAACCATGGAGAGAA | Transducin (JQ711533) | 60 | 50 | 205 | 60 |
| Transd Rev | TTTTCCAAAGAACCCACCAG |  | 59 | 45 |  |  |
| Ubiquitin For | GTGGATCGTCTGGATTTGGA | Ubiquitin-coniugating enzyme (JQ429798) | 60 | 50 | 156 | 60 |
| Ubiquitin Rev | TCCGGTTCCTCACCAAAATA |  | 60 | 45 |  |  |
| UP1-For | GCATTGGTCAGTATAATGCATCCT | Unknown protein 1 (JQ711535) | 58 | 42 | 110 | 58 |
| UP1-Rev | GGAAGCAGCAACACTTTTTCAA |  | 58 | 41 |  |  |
| UP2-For | CGACGAACTTCACTCAGCCA | Unknown protein 2 (JQ711536) | 59 | 55 | 110 | 59 |
| UP2-Rev | TCCGCCTTCTCTTCACTGATC |  | 58 | 52 |  |  |
